# Selective Inhibitors of Protozoan Protein *N*-myristoyltransferases as Starting Points for Tropical Disease Medicinal Chemistry Programs

**DOI:** 10.1371/journal.pntd.0001625

**Published:** 2012-04-24

**Authors:** Andrew S. Bell, James E. Mills, Gareth P. Williams, James A. Brannigan, Anthony J. Wilkinson, Tanya Parkinson, Robin J. Leatherbarrow, Edward W. Tate, Anthony A. Holder, Deborah F. Smith

**Affiliations:** 1 Worldwide Medicinal Chemistry, Pfizer Worldwide Research and Development, Kent, United Kingdom; 2 High Throughput Screening Center of Emphasis, Pfizer Worldwide Research and Development, Kent, United Kingdom; 3 Department of Chemistry, University of York, York, United Kingdom; 4 Opportunities for Partnership in Medicine, Pfizer Worldwide Research and Development, Kent, United Kingdom; 5 Institute of Chemical Biology, Department of Chemistry, Imperial College, London, United Kingdom; 6 Division of Parasitology, Medical Research Council National Institute for Medical Research, London, United Kingdom; 7 Department of Biology, University of York, York, United Kingdom; Northeastern University, United States of America

## Abstract

Inhibition of *N*-myristoyltransferase has been validated pre-clinically as a target for the treatment of fungal and trypanosome infections, using species-specific inhibitors. In order to identify inhibitors of protozoan NMTs, we chose to screen a diverse subset of the Pfizer corporate collection against *Plasmodium falciparum* and *Leishmania donovani* NMTs. Primary screening hits against either enzyme were tested for selectivity over both human NMT isoforms (*Hs*1 and *Hs*2) and for broad-spectrum anti-protozoan activity against the NMT from *Trypanosoma brucei*. Analysis of the screening results has shown that structure-activity relationships (SAR) for *Leishmania* NMT are divergent from all other NMTs tested, a finding not predicted by sequence similarity calculations, resulting in the identification of four novel series of *Leishmania*-selective NMT inhibitors. We found a strong overlap between the SARs for *Plasmodium* NMT and both human NMTs, suggesting that achieving an appropriate selectivity profile will be more challenging. However, we did discover two novel series with selectivity for *Plasmodium* NMT over the other NMT orthologues in this study, and an additional two structurally distinct series with selectivity over *Leishmania* NMT. We believe that release of results from this study into the public domain will accelerate the discovery of NMT inhibitors to treat malaria and leishmaniasis. Our screening initiative is another example of how a tripartite partnership involving pharmaceutical industries, academic institutions and governmental/non-governmental organisations such as Medical Research Council and Wellcome Trust can stimulate research for neglected diseases.

## Author Summary

Inhibition of *N*-myristoyltransferase has been validated pre-clinically as a target for the treatment of fungal and trypanosome infections, using species-specific inhibitors. In order to identify inhibitors of protozoan NMTs, we chose to screen a diverse subset of the Pfizer corporate collection against *Plasmodium falciparum* and *Leishmania donovani* NMTs. Primary screening hits against either enzyme were tested for selectivity over both human NMT isoforms (*Hs*NMT1 and *Hs*NMT2) and for broad-spectrum anti-protozoan activity against the NMT from *Trypanosoma brucei*. We have identified eight series of protozoan NMT inhibitors, six having good selectivity for either *Plasmodium* or *Leishmania* NMTs over the other orthologues in this study. We believe that all of these series could form the basis of medicinal chemistry programs to deliver drug candidates against either malaria or leishmaniasis. Our screening initiative is another example of how a tripartite partnership involving pharmaceutical industries, academic institutions and governmental/non-governmental organisations such as the UK Medical Research Council and Wellcome Trust can stimulate research for neglected diseases.

## Introduction

Protozoan parasites are major causative agents of global infectious diseases that affect millions of people in tropical and sub-tropical regions of the world [1]. In the absence of effective vaccination strategies, treatment for many of these infections depends on chemotherapy and is reliant on “old” drugs that have often been in use for long periods; were originally developed for other types of disease; give rise to increasing levels of microbial resistance; and often show unacceptable levels of toxicity. There is a pressing need for new therapeutics that can be targeted to the populations that need them. This work focuses on two groups of diseases: the leishmaniases (caused by species of the kinetoplastid parasite, *Leishmania*) and malaria (caused by species of the apicomplexan parasite, *Plasmodium*).

In the case of the leishmaniases (a spectrum of diseases associated with immune dysfunction), there are estimated to be 1.5–2 million new cases each year in 88 countries around the globe, with 350 million people at risk and a disease burden of ∼2.4 million disability-adjusted life years [2], [3]. Clinical symptoms, ranging from the disfiguring skin lesions of cutaneous leishmaniasis to the often fatal visceral leishmaniasis (VL – predominantly caused by *Leishmania donovani*) are exacerbated in children and immuno-compromised patients, such as those diagnosed as HIV positive. Pentavalent antimonials have been the first-line treatment for VL since the 1930s but these compounds are toxic, with resistance an increasing problem in the Indian sub-continent [4]. While significant progress has been made in the last 10 years, with the approval of lipid formulations of amphotericin B, miltefosine and paromomycin, none of these has been developed by rational design, and resistance may still be a problem. There is no effective vaccine available, although vaccination should theoretically be possible against these infections that can “self-heal” in their most benign states. Thus new strategies for vaccination and chemotherapy, particularly for visceral leishmaniasis (VL), remain an urgent international priority [5].

Malaria remains one of the most important infectious diseases of the developing world. There were an estimated 216 million episodes in 2010, resulting in between 650,000 and 1.2 million deaths, according to two recent reports, with over 90% occurring in Africa [6], [7]. Although *P. falciparum* accounts for 75% of malaria cases and most of the deaths, *P. vivax* is also a significant problem in South East Asia, and South and Central America [8]. There is an urgent need to develop new drugs with rapid efficacy, minimal toxicity and low cost to replace chloroquine and pyrimethamine-sulphadoxine (available as Fansidar), which are failing rapidly due to resistance in *P. falciparum* [9]–[11]. The use of artemisinin and its derivatives, such as artesunate or artemether, which have good efficacy but very short half-lives, together with longer acting agents such as amodiaquine or lumefantrine in artemisinin-based combination therapy (ACT), offers some respite. However these drugs are expensive, development of resistance is an ever present possibility, and new effective drugs will be required [12]. In addition it is important to develop new drugs that also target blood stage sexual forms of the parasite to prevent transmission, particularly of drug resistant parasites [13]. At a recent Malaria Forum, the Gates Foundation [14] called for the eradication of malaria. This goal will realistically only be achieved by supplementing current control methods with the development of vaccines and new drugs.

The disease statistics above make a compelling case for accelerated drug development, which should be facilitated by recent rapid progress in genome sequencing and subsequent post-genomic strategies for target identification [12], [15]. Numerous targets have been assessed, including pyrimidine biosynthesis [16], nucleotide signalling [17], kinase pathways [18] and lipidation [19]. Inhibition of prenylation has shown potential to treat malaria and more recently, lipid modification through inhibition of *N*-myristoylation has shown encouraging progress.

Co–translational myristoylation is catalysed by the monomeric enzyme myristoyl CoA: protein *N*-myristoyltransferase (NMT) in all eukaryotes and has been shown to be essential for viability in fungi and protozoa, through both genetic studies and by chemical inhibition of *Candida albicans* NMT [20], [21] and *Trypanosoma brucei* NMT (*Tb* NMT) [22], [23]. Our earlier work on the NMTs of *T. brucei* (the kinetoplastid causative agent of African sleeping sickness) [24]–[26], *L. major* [26] (causative agent of cutaneous leishmaniasis) and *P. falciparum (Pfal)* [27], [28], identified NMT as a suitable candidate for drug development against the diseases caused by these protozoan organisms [29], [30].

**Figure 1:**
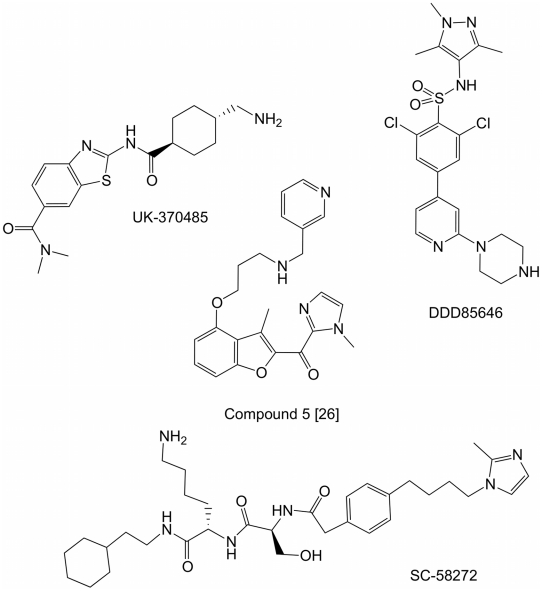
Structures of representative, previously reported NMT inhibitor series. Three distinct series of *C.albicans* NMT inhibitors, exemplified by SC-58272, UK-370485 and compound 5 [31] , and a series of inhibitors of both *T.brucei* and *L.donovani* NMT (e.g. DDD85646), have been reported in the chemical literature. Co-crystal structures with their respective targets have shown that each inhibitor binds in the same region of the binding site as the substrate peptides (Figure 2).

NMT from *Tb* has already been demonstrated to be a druggable target using small molecules (Figure 1) [22], [23]. In addition, NMTs from fungal species e.g. *C. albicans* and *Aspergillus fumigatus* have also been long-standing targets within the pharmaceutical industry and several inhibitor series have been reported [20], [21], [31]. With the exception of the Searle series, which are peptidomimetics based on the *C. albicans Arf* protein, all other published NMT inhibitor series were obtained by high-throughput screening.

**Figure 2:**
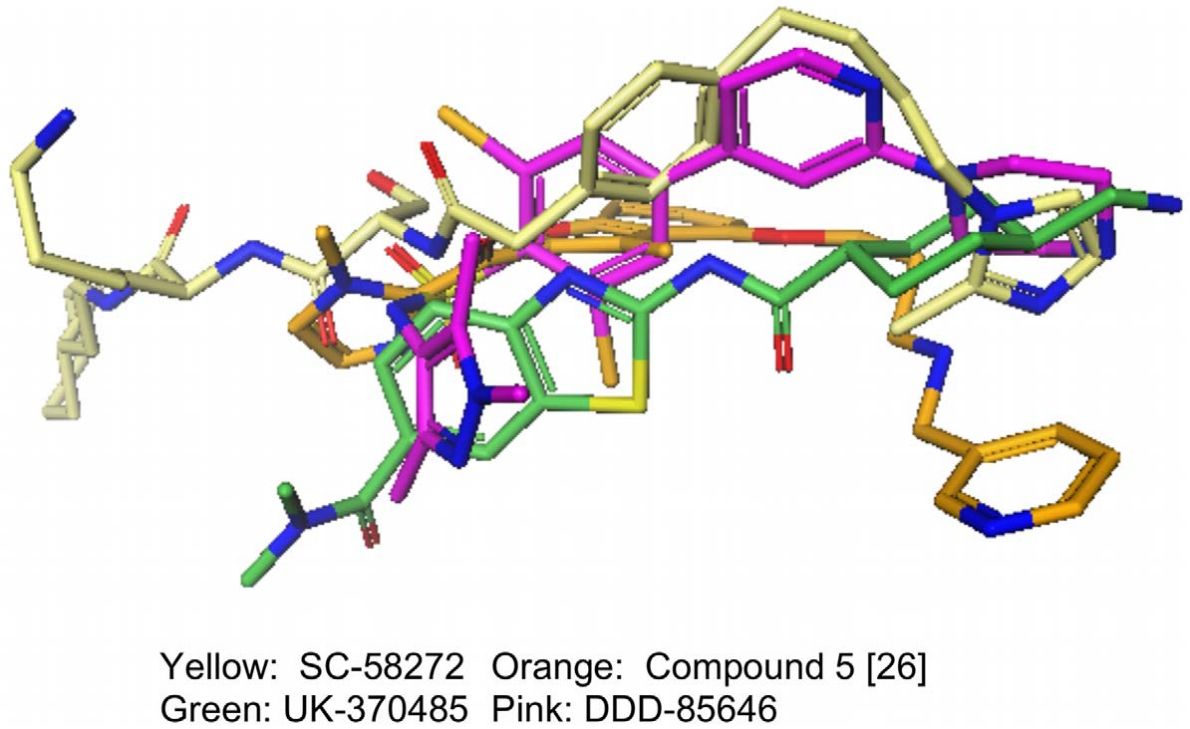
Overlay of structures of inhibitors (see **Figure 1**) based on alignment of binding site residues. The crystal structural information for NMTs from fungal (*C. albicans, Saccharomyces cerevisiae*), human and protozoan (*L. major and L donovani*) NMTs was used as the basis for modelling work. The binding-site residues were aligned to create a view of the occupation of the NMT binding sites across species (based on 1iyl, 2wsa, 1iyk, and an unpublished structure for UK-370485 in *C. albicans* NMT). Images were created using the Pfizer molecule-modelling package MoViT and the inhibitors colored (Yellow: SC-58272: Orange: Compound 5 [31]; Green: UK-370485; Pink: DDD85646). Despite binding in the same region of their respective NMT, each compound occupies a different sub-region, which supported the case for high-throughput screening as a source of novel NMT inhibitor series.

Structures of representative inhibitors bound to their respective NMT targets are available and each shows inhibitors binding in the same region as the substrate peptide. A wide variety of proteins are reported or predicted as substrates for myristoylation based on an N-terminal consensus sequence for substrates (GXXXSK/L) [32]. The broad scope of amino acids that are tolerated close to the amino terminal is a reflection of a relatively wide channel, which can be used to rationalise the diversity of the inhibitor structures. The published molecular structures from the fungal and *T. brucei* NMT programs were used to overlay the ligands in a common co-ordinate frame, (Figure 2), and could be used to rationalise the observed selectivity e.g. for fungal *vs.* protozoan NMTs.

The presence of compounds from the legacy Searle and Pfizer fungal NMT programs, within the Pfizer corporate collection, has made screening of the Pfizer file an attractive option for the identification of inhibitors of *Plasmodium* and *Leishmania* NMTs [33]–[35]. In this study, we extend the scope of screening to sample the diversity of the entire Pfizer compound file.

Due to time and cost constraints, we decided to limit our primary screening campaign to ∼150,000 compounds from the Pfizer Global Diverse Representative Set [36], against both *Pfal* NMT and *L. donovani* (*Ldon*) NMT since these protozoan NMTs are sufficiently dissimilar at the sequence level to warrant separate screens. Activity of hit compounds would be confirmed in replicated dose-response assays to generate a preliminary SAR for each target.

All mammalian systems studied to date have been shown to have two NMT genes, *nmt1* and *nmt2* [37]. *Hs*NMT1 has been shown to be essential for embryonic development in mice, whilst the physiological role of *Hs*NMT2 is currently unknown [38]. However, a number of oncogenic proteins are substrates of human NMTs, and certain viruses and bacteria also exploit host NMTs to myristoylate their own proteins. Consequently, human NMTs have been proposed as targets for the treatment of cancer and viral infections [29]. To date, there have been no conclusive reports on the potential toxicity of mammalian NMT inhibition *in vivo*, but in view of the essential role of NMT in mammalian development, selectivity over human NMTs is desirable. All hits from this study should therefore be screened in dose–response assays against both human isoforms *Hs*NMT1 & *Hs*NMT2 (*Hs*1 *& Hs*2). Hits might also be tested against the NMT from *Tb*, since activity against multiple protozoan NMTs would be an advantage, provided selectivity over human NMTs could be achieved.

## Methods

### *P. falciparum* and *L. donovani* NMT screening

Recombinant *P. falciparum* [39] (Swiss-Prot Q81LW6) and *L. donovani* NMTs [40] (EMBL accession number FN555136) were produced at the University of York, using established protocols. An assay format based on scintillation proximity technology to monitor enzyme activity was used as previously described [27], [30]. This radioactive format provides a quantification of the *N*-myristoylation of the synthetic peptide CAP5.5 (derived from the *N*-terminus of the *T. brucei* CAP5.5 protein) [41] as used in [22], [24], and was modified to a 384-well plate screening format with a final volume of 40 µl.

The Pfizer Global Diverse Representative Set [36] consisting of 150,000 compounds was screened at 20 µM final assay concentration. Assay plates were prepared by dispensing 0.1 µL of compound dissolved in DMSO using the Vario system (Cybio) into white 384-well plates (Greiner Bio-One). The assay buffer consisted of 30 mM Tris-HCl pH 7.4, 0.5 mM EGTA, 0.5 mM EDTA, 0.1% Triton X-100 and 1.25 mM DTT.

Enzyme solution containing the appropriate NMT (*Pfal* at 3.7 nM final assay concentration, *Ldon* at 0.84 nM) diluted in assay buffer was added to each well in a volume of 10 µl using a Multidrop Combi dispenser (Thermo Scientific). Plates were incubated for 15 min at room temperature (RT) before the addition of substrate solution, consisting of cold myristoyl CoA (Sigma), ^3^H-myristoyl CoA (ARC) and CAP5.5 at the respective final assay concentrations of 54 nM, 8.5 nM and 250 nM.

The reaction was initiated by adding 10 µL of the substrate mixture to each well containing the pre-incubated enzyme/compound mixture, and allowed to proceed at RT for 80 min. The reaction was quenched by the addition 20 µl of stop solution to each well. Stop solution consisted of 0.75 M MgCl_2_ (Sigma), 0.1 M phosphoric acid (Sigma) and 1 mg/ml of streptavidin-coated polystyrene SPA beads (Perkin-Elmer). Plates were sealed using topseals (Perkin-Elmer) and left overnight to allow the bead solution to settle. Radioactive counts were measured with an integration time of 300 sec per plate using the Leadseeker Generation 4 plate reader (GE Healthcare). Each plate included a positive control of 1 µM final assay concentration of DDD85646 [22] and 1% DMSO as a negative control. Hit compounds were further titrated using a through-plate IC50 format with a top concentration of 80 µM, and 12 points with a 1∶2 dilution step. The data were analysed using Pfizer SIGHTS software and visualised using Spotfire software (TIBCO).

### *T. brucei* and Human NMT screening

Additional dose-response assays using the *T brucei* NMT [22], [24] (EMBL FN554973) and human enzymes *Hs*NMT1 [39](Swiss-Prot P30419) and *Hs*NMT2 [39](Swiss-Prot O60551), produced at the University of York using established protocols, were carried out to assess the activity spectrum of hit compounds. The assay format used was the same as above, however the respective concentrations of the enzymes were 4.8 nM, 2.6 nM and 2.6 nM, respectively. Substrate concentrations were 108 nM myristoyl CoA, 17 nM ^3^H-myristoyl CoA and 500 nM CAP5.5 (final assay concentration) for all three specificity screens. The stop solution had a reduced SPA bead concentration of 0.5 mg/ml. The *Tb* NMT screen reaction incubation was 60 min, while the *Hs*1 and *Hs*2 reaction incubation was 15 min. The same dose-response format of 80 µM top concentration and a 12-point curve, with a 1∶2 dilution step was used.

## Results

### Analysis of Previous NMT Inhibitors

While the reported NMT inhibitors all occupy the substrate peptide-binding site, structural studies on *C. albicans* NMT have shown that distinct inhibitor series exploit different interactions with the protein (Figure 2). Consequently, we envisaged that a set of high-throughput screening hits would be likely to represent a number of different binding modes. We anticipated that a different binding mode might result in a different selectivity profile against the set of NMTs in this study. In the case of the recently reported *Tb*NMT inhibitors, the lead compound is reported to have equivalent potency against both *Hs*NMT1 and *L. major* NMT [22]. In contrast, our previous work had shown that even a single residue change in the binding region of *C. albicans* NMT was sufficient to result in a three-hundred-fold loss of enzyme affinity [35].

**Figure 3:**
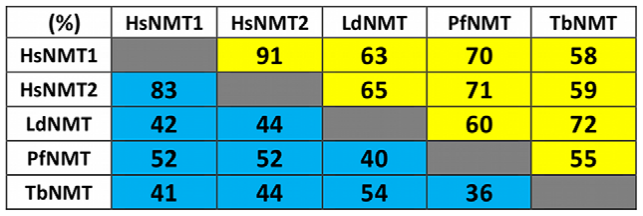
Comparison of protein identity and similarity for the NMT isoforms in this study. Figure showing the percentage sequence identity (Blue) and sequence similarity (Yellow) as defined by BLAST between the NMT proteins from *L. donovani* (*Ldon*NMT, EMBL accession number FN555136), *P. falciparum* (*Pfal*NMT, Swiss-Prot Q81LW6), *T. brucei* (*Tb*NMT, EMBL FN554973) and the catalytic domains of Human NMT isoforms *Hs*1NMT1 (Swiss-Prot P30419) and *Hs*2NMT (Swiss-Prot O60551.

We hypothesised that the number of global residue changes between the NMT proteins would correlate with increasingly divergent SAR for the various NMT proteins in this study. Publically available sequence information was used to calculate pairwise similarity and identity between the NMT proteins in the study (Figure 3). Although an ideal compound would have broad-spectrum activity against the protozoan NMTs but would lack activity against both human NMTs, our analysis suggested that this could difficult to achieve, unless some distinct differences in the binding sites could be exploited. The sequence similarities suggest that it should be more likely for *Ldon* NMT hits to be selective over *Pfal* and *Hs*NMTs, but that *Pfal*NMT hits would be less likely to achieve selectivity over *Hs*NMTs.

### *P. falciparum* and *L. donovani* NMT screening

**Figure 4:**
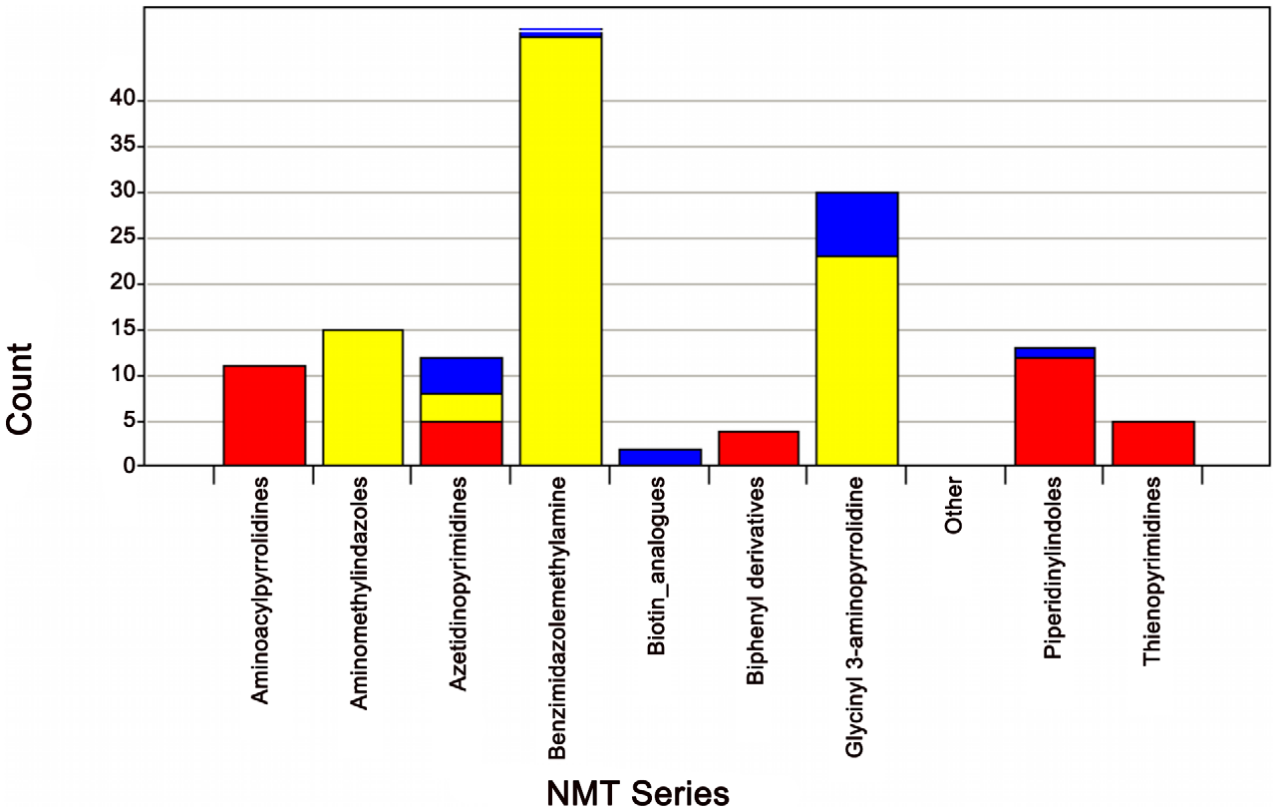
Bar chart showing numbers of active compounds in each chemical series. Compounds with >40% activity against either *Ldon* [red], *Pfal* [yellow] or both NMT targets [blue], were sub-divided by chemical series. Most of the series were selective for one NMT orthologue. Molecules active against both targets were often highly active against one NMT and weakly active against the other primary screening target.

**Figure 5:**
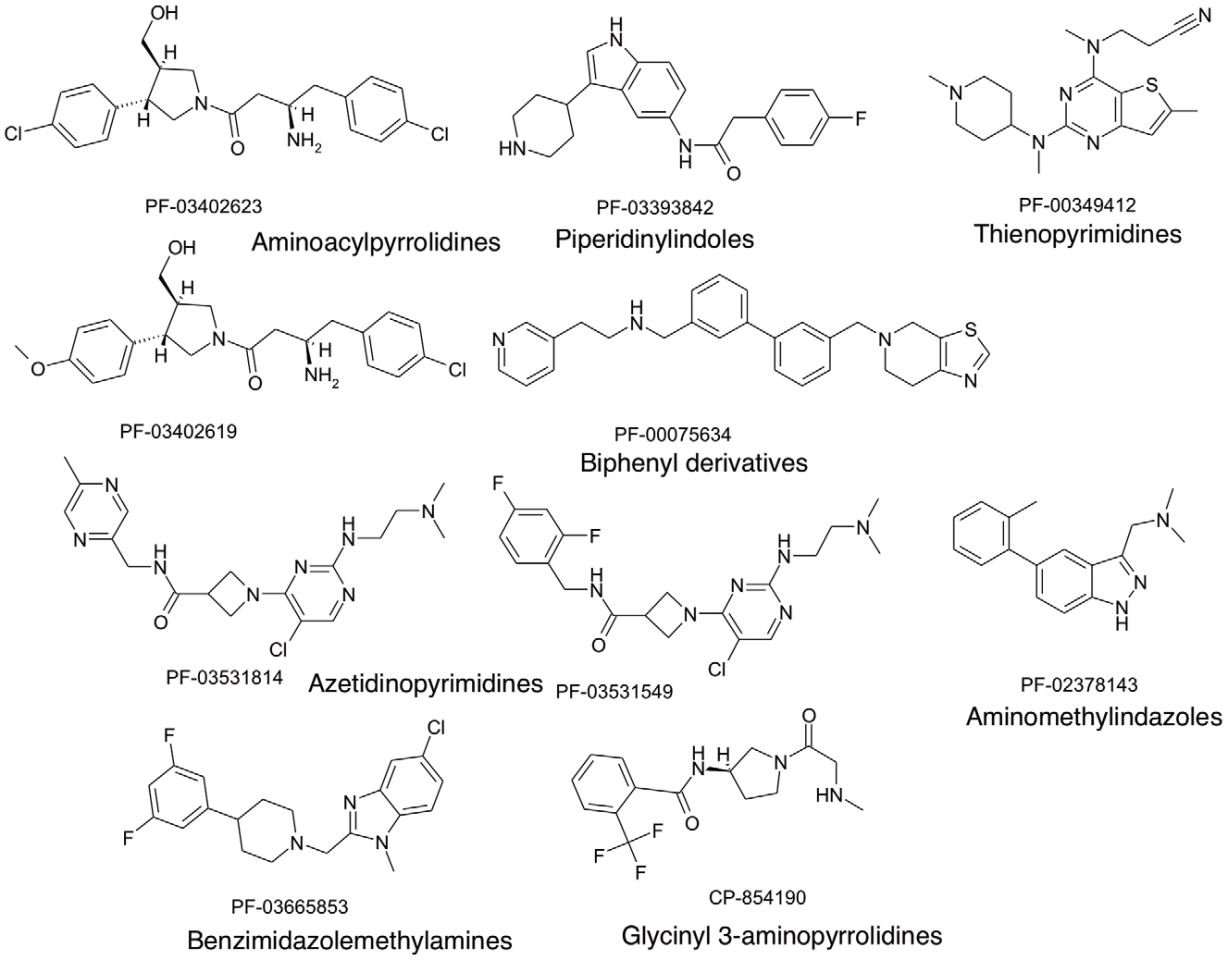
Structures of representative hit compounds from NMT screening. Triage of the primary screening hits made use of local hit rate analysis; ligand efficiency against the primary target; selectivity in the primary screen; and broader NMT selectivity. Eight of the series were judged to contain lead compounds capable of further development towards new medicines to treat malaria or leishmaniasis. Table 1 summarises the primary screen activity of selected preferred examples from each series and a further series representative when analogue screening identified a superior compound. The broader NMT screening profile of each compound is detailed in Table 2.

The *Pfal* and *Ldon* HTS generated Z′ scores of 0.84 and 0.78, respectively, indicating that the screening results were of excellent quality [42]. In order to capture all potential actives, compounds conferring above 40% inhibition were considered to be hits, giving an overall hit rate of 0.8% and 0.4%, respectively, with 0.1% of the compounds being classified as hits against both NMTs. Since the Pfizer file consists of discrete clusters of compounds, either from parallel synthesis libraries or medicinal chemistry series, we used this series of origin definition, rather than a clustering algorithm, to label the series. Encouragingly, hit series tended to inhibit one of the target NMTs selectively. All hits were progressed to dose-response assays using a top concentration of 80 µM. Hits were classified as confirmed if they gave a measured IC50 of <5 µM against either enzyme. They were further sub-divided into selective inhibitors of either NMT or both enzymes (Figure 4). The activities of the most potent example from selected, novel series of NMT inhibitors is summarised in Table 1 (see Figure 5 for structures of the hit molecules). Several other series identified in the primary screen were discarded due to low potency, lack of selectivity or due to on-going interest for other Pfizer programs.

**Table 1:** Primary dose-response profile of representative hits from each chemical series.

Series	Compound ID	# hits	*Pfal* LHR	*Pfal* IC50	*Pfal* LE	*Ldon* IC50	*Pfal/Ldon* Sel
Glycinyl 3-aminopyrrolidine	CP-854190	30	0.03	1.72	0.34	38.7	22.5
Benzimidazolemethylamine	PF-03665853	47	0.04	0.91	0.32	>80	>88
Aminomethylindazoles	PF-02378143	15	0.11	2.54	0.38	65.4	25.8
Azetidinopyrimidines	PF-03531549	9	0.04	1.7	0.27	1.2	0.7
Azetidinopyrimidines	PF-03531814	9	n/a	0.482	0.31	72	150

Series	Compound ID	# hits	*Ldon* LHR	*Ldon* IC50	*Ldon* LE	*Pfal* IC50	*Ldon/Pfal* Sel
Aminoacylpyrrolidines	PF-03402619	12	0.07	0.901	0.29	>80	>88
Aminoacylpyrrolidines	PF-03402623	12	n/a	0.093	0.35	71.9	770
Piperidinylindoles	PF-03393842	13	0.21	0.102	0.37	9.0	88.5
Thienopyrimidines	PF-00349412	5	0.14	0.482	0.34	39.6	82
Biphenyl derivatives	PF-00075634	4	0.09	0.158	0.29	>80	>505

# Hits are compounds in series with activity <5 µM against the respective NMT. LHR = Local hit rate @ 0.4 similarity to indicate the proportion of similar compounds with activity in the primary screen. IC50 data are the mean of 3 separate experiments (in µM). These data were used to calculate ligand efficiency (LE) for each compound against their target NMT, and to derive selectivity (Sel) values for the primary screening targets.

As our initial high-throughput screen sampled only around 5% of the Pfizer screening collection, we sought to expand our hit identification through further analogue screening. Several approaches were employed including substructure searching, similarity searching and by the creation of Bayesian activity models based on the primary screening data [43]. However, the most successful tool used local hit rates to identify hit rich series (Table 1) [44]. In two series, we were able to identify more potent hits from further screening (compounds in Table 1 without local hit rate value). Further series with low local hit rate values tended to be weakly active on repeat testing.

When selecting series for further follow-up, we focused on compounds with selective activity against either NMT and with a ligand efficiency (LE) of greater than 0.30 [45]–[47]. Unlike gene families such as GPCR antagonists or kinase inhibitors, we had insufficient data on transferase enzymes to guide our expectations for the level of LE that might be attained in a drug candidate. However, the level we achieved is consistent with those achieved by peptidomimetic protease inhibitors. Consequently, we believe that all of the series exemplified have sufficiently high ligand efficiency to warrant further development as lead compounds.

### *T. brucei* and Human NMT screening

2066 compounds were selected for wider profiling against a panel of NMT orthologues, based on either activity in the primary screen or chemical similarity, to generate a pharmacological profile of each NMT in the study, relative to each other. The compound set included three biotin derivatives, which were known to be false positives in this assay format and were shown to have equivalent activity against each NMT orthologue, thus acting as additional positive controls.

**Figure 6:**
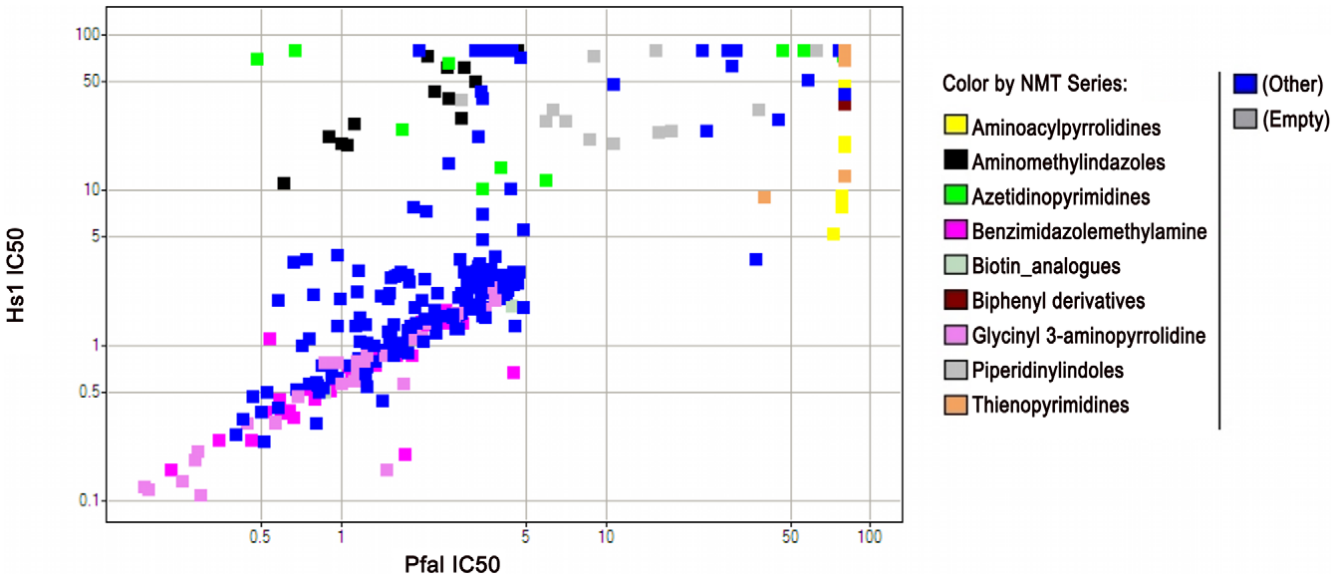
Plot of dose-response activity against *Pfal* vs. *Hs*1 NMTs for primary screening hit and analogue screening set. Broader selectivity screening was carried out using 5 NMT orthologues and a set of 2066 compounds. A plot showing the potency of each compound against *Pfal* and *Hs*1 showed that most compounds lacked selectivity. All compounds included in the broad selectivity screening were labelled with their chemical series. The series with potential for further follow-up are disclosed in this paper. The other series were discarded due to lack of enzyme activity or NMT-selectivity. Two series stood out from the general trend; representative structures for these series are shown in Figure 5. Two additional series provided potent, non-selective inhibitors, which may also be useful as starting points for medicinal chemistry programs.

Two key findings emerged from this study. Firstly, we found that the vast majority of the *Pfal* NMT hit series were equipotent against both human NMTs (Figure 6 data shown for *Hs*1). Only two series (azetidinopyrimidines and aminomethylindazoles) showed a divergent SAR for *Plasmodium vs.* human NMTs (see Table 2 for data on representative compounds), and in the case of the azetidinopyrimidine series, selectivity was only observed in some derivatives.

**Table 2:** Activity of lead compounds against all NMT orthologues in this study.

Series	Compound ID	*Pfal* IC50	*Ldon* IC50	*Tb I*C50	*Hs*1 IC50	*Hs*2 IC50
Glycinyl 3-aminopyrrolidine	CP-854190	1.72	38.7	>80	0.57	1.59
Benzimidazolemethylamine	PF-03665853	0.91	>80	35.4	0.51	0.56
Aminomethylindazoles	PF-02378143	2.54	65.4	>80	38.9	56.3
Azetidinopyrimidines	PF-03531549	1.7	1.2	52.7	11.5	16.1
Azetidinopyrimidines	PF-03531814	0.48	72	>80	70.0	76.0
Aminoacylpyrrolidines	PF-03402619	>80	0.90	9.9	47.2	63.6
Aminoacylpyrrolidines	PF-03402623	71.9	0.093	1.5	5.2	14.7
Piperidinylindoles	PF-03393842	9.0	0.10	35.9	73.2	64.1
Thienopyrimidines	PF-00349412	39.6	0.48	56.2	9.0	11.5
Biphenyl derivatives	PF-00075634	>80	0.16	>80	36.1	26.7

All data are in µM. *Pfal* and *Ldon* NMT values are the mean of 3 determinations, *Hs*1, *Hs*2 and *Tb* NMT IC50 values were from a single determination.

**Figure 7:**
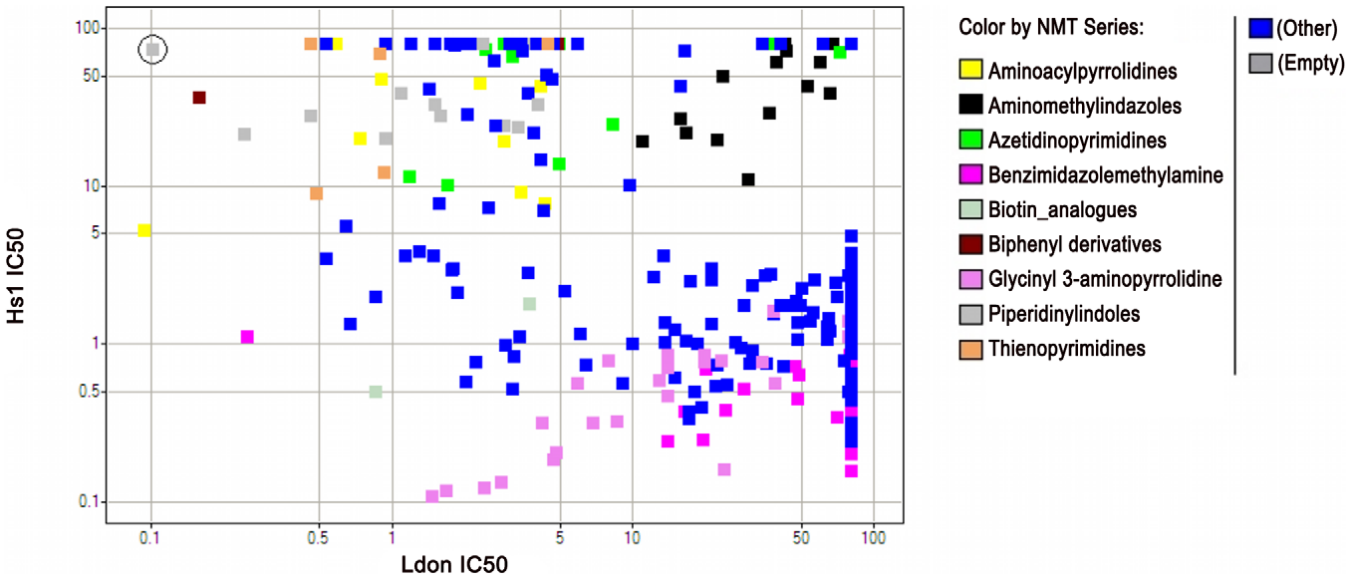
Plot of dose-response activity against *Ldon* vs. *Hs*1 NMTs for primary screening hit and analogue screening set. In contrast to Figure 6, a plot of potency against *Ldon* and *Hs*1 NMTs showed that activity did not correlate. Excellent selectivity was observed for several chemical series. The structures of representative members of four series are given in Figure 5.

Secondly, and in contrast to the *Pfal* findings, there was no correlation between activity against *Ldon* NMT and human NMTs (Figure 7 for *Hs*1). In addition, the *Leishmania* NMT hits were selective over both other protozoan NMTs (Table 2). Our study also provided an insight into the selectivity profiles between human NMTs and Tb NMT (Figures S1 and S2).

## Discussion

We have exploited knowledge of the essential function of NMT in protozoa, and harnessed the strength of the Pfizer compound collection, to initiate a drug discovery program for this target in *Plasmodium* and *Leishmania*. 150,000 compounds were screened against *Pfal* and *Ldon* NMTs. Based on initial modelling studies and sequence alignments, we opted to assess the quality of the hits through selectivity assays against both *Hs* NMT isoforms and against *Tb* NMT, to assess their potential to deliver broad-spectrum anti-protozoan NMT activity. Our results suggest that this objective is unlikely to be successful since we found that selectivity between the protozoan NMTs is readily achievable but that most *Pfal* NMT inhibitors are equipotent against the human isoforms. These results suggest that *Pfal*/human NMT selectivity might be a barrier to drug discovery for this target, unless inhibition of mammalian NMTs is shown to have no toxicological effects. However, we discovered two novel series with sufficient selectivity to encourage further medicinal chemistry follow-up. These findings suggest that some compound series are binding in regions that differ between *Pfal* and human NMTs. If these differences can be rationalised, ideally through obtaining co-crystal structures with *Plasmodium* NMT, even non-selective hits could be of potential value as starting points for medicinal chemistry programs.

Despite a lower initial screening hit rate, we found four series of *Ldon* NMT inhibitors with good to excellent selectivity over all other NMTs in our panel. While this result was in line with our predictions for human and *Pfal* NMTs, the separation of *Ldon* and *Tb* activity was unexpected. This result contrasts with those previously reported for another series of *Tb* NMT inhibitors, therefore suggesting that all of the series we have identified bind in a different region of the binding pocket [23]. This observation will be tested through further structural studies with *Leishmania* NMT. It also appears that *Ldon* NMT is more susceptible to potent inhibition, perhaps an indication that the peptide-binding site is smaller than for the other NMTs in this study. Based on the results we describe, it is also likely that high throughput screening of the Pfizer file against other NMT orthologues would have yielded further selective series. These results underline the benefit of high-throughput screening of a diverse compound collection to discover novel protozoan NMT inhibitors, as an alternative to a “piggy-backing” approach [19].

This early drug discovery collaboration was facilitated by grants from the Wellcome Trust and MRC, which demonstrates the power of such public private partnerships in bringing together the drug discovery expertise of pharmaceutical companies and the detailed target knowledge from academia to accelerate drug discovery for neglected tropical diseases. Our most promising compounds are disclosed to accelerate the pace of drug development for malaria and leishmaniasis. These hits represent excellent starting points for a future medicinal chemistry program, although they have yet to be tested in whole cell assays against their target organisms. Our future work on *Pfal* and *Ldon* NMTs will focus on the translation of enzyme inhibition into functional activity.

## Supporting Information

Figure S1 **Plot of dose-response activity against** ***Ldon*** **vs.** ***Tb*** **NMTs for primary screening hit and analogue screening set.** While data reported for the series exemplified by DDD85646 showed equal potency against both *Ldon* and *Tb* NMTs, a plot of potency against *Ldon* and *Tb* NMTs showed that activity did not correlate across the wider screening set The combination of these data with those in previous Figures suggests that broad-spectrum anti-protozoan NMT inhibition is unlikely to be achievable.
(TIF)

Figure S2 **Plot of dose-response activity against** ***Hs1*** **vs.** ***Hs*** **2 NMTs for primary screening hit and analogue screening set.** Data for the wider screening set against both human NMTs showed an excellent correlation. Since the human NMTs have the most similar sequences of all of the orthologues in this study, there is no evidence from this analysis that selective inhibition will be achievable.
(TIF)
