# Supplementary figures and images for: Selective Inhibitors of Protozoan Protein N-myristoyltransferases as Starting Points for Tropical Disease Medicinal Chemistry Programs
Source: PLoS Negl Trop Dis. 2012 Apr 24;6(4):e1625. doi: 10.1371/journal.pntd.0001625 (PMC3335879; doi:10.1371/journal.pntd.0001625)

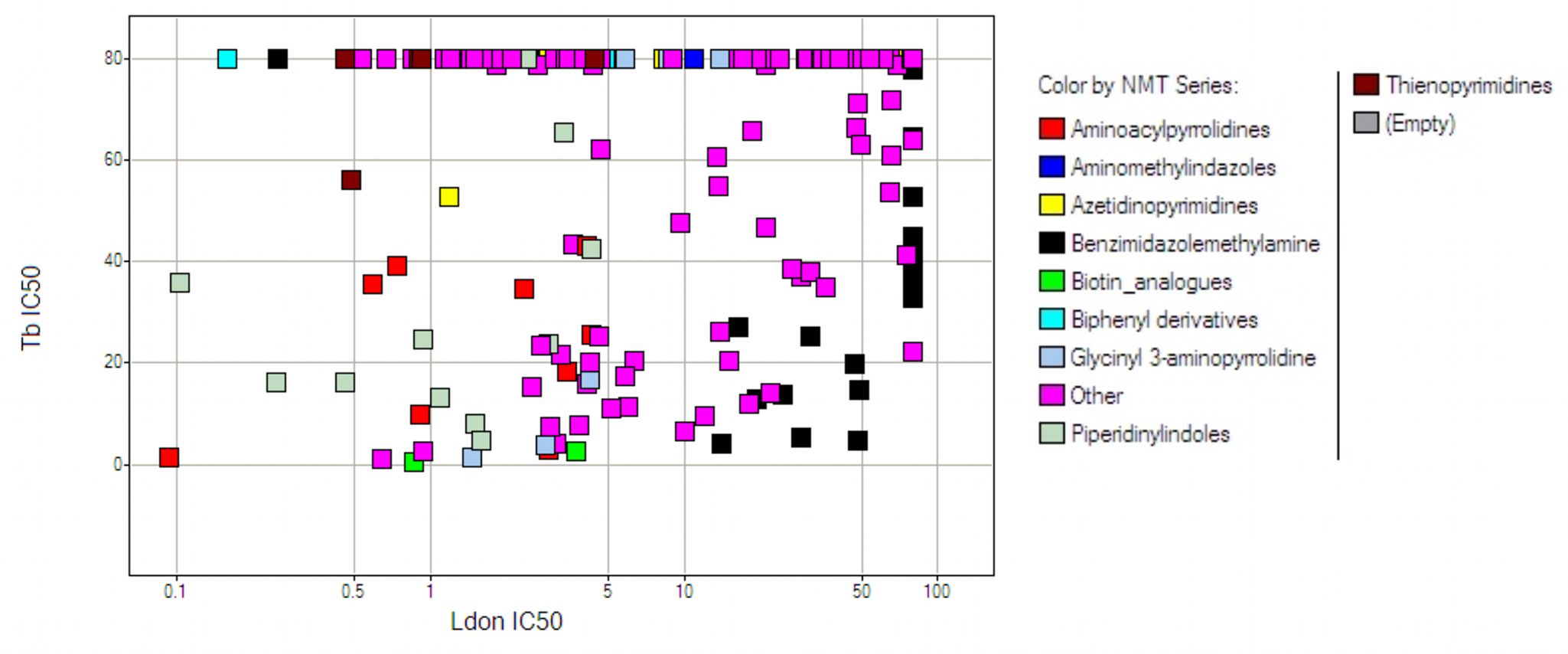

Supplement: Figure S1 — Plot of dose-response activity against Ldon vs. Tb NMTs for primary screening hit and analogue screening set. While data reported for the series exemplified by DDD85646 showed equal potency against both Ldon and Tb NMTs, a plot of potency against Ldon and Tb NMTs showed that activity did not correlate across the wider screening set The combination of these data with those in previous Figures suggests that broad-spectrum anti-protozoan NMT inhibition is unlikely to be achievable. (TIF) [file pntd.0001625.s001.tif]

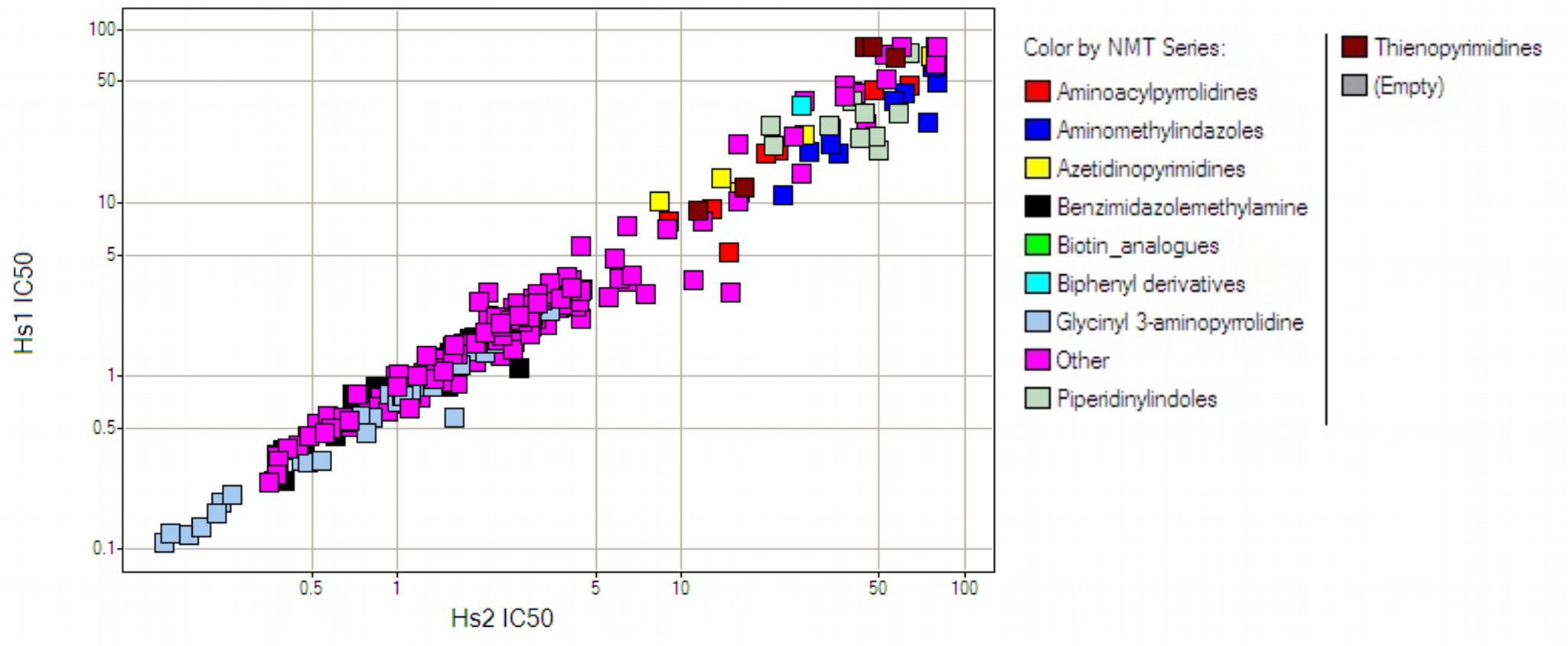

Supplement: Figure S2 — Plot of dose-response activity against Hs1 vs. Hs 2 NMTs for primary screening hit and analogue screening set. Data for the wider screening set against both human NMTs showed an excellent correlation. Since the human NMTs have the most similar sequences of all of the orthologues in this study, there is no evidence from this analysis that selective inhibition will be achievable. (TIF) [file pntd.0001625.s002.tif]
